# Dental Pulp Testing: A Review

**DOI:** 10.1155/2009/365785

**Published:** 2009-11-12

**Authors:** Eugene Chen, Paul V. Abbott

**Affiliations:** School of Dentistry, The University of Western Australia, Nedlands, Perth, WA 6009, Australia

## Abstract

Dental pulp testing is a useful and essential diagnostic aid in endodontics. Pulp sensibility tests include thermal and electric tests, which extrapolate pulp health from sensory response. Whilst pulp sensibility tests are the most commonly used in clinical practice, they are not without limitations and shortcomings. Pulp vitality tests attempt to examine the presence of pulp blood flow, as this is viewed as a better measure of true health than sensibility. Laser Doppler flowmetry and pulse oximetry are examples of vitality tests. Whilst the prospect is promising, there are still many practical issues that need to be addressed before vitality tests can replace sensibility tests as the standard clinical pulp diagnostic test. With all pulp tests, the results need to be carefully interpreted and closely scrutinised as false results can lead to misdiagnosis which can then lead to incorrect, inappropriate, or unnecessary treatment.

## 1. Introduction

Dental pulp tests are investigations that provide valuable diagnostic and treatment planning information to the dental clinician [1]. If pathosis is present, pulp testing combined with information taken from the history, examination, and other investigations such as radiographs leads to the diagnosis of the underlying disease which can usually be reached relatively easily. 

From the late 1970s until the 1990s, applications of pulp tests in different areas of clinical dentistry were met with varying degrees of success [1, 2]. This could be interpreted that, to date, the notion of the ideal diagnostic test [3] is still to be realised. From a technical perspective, all current pulp tests have shortcomings, especially in terms of accuracy, reliability, and reproducibility in a given diagnostic challenge. In addition, the correct application of the pulp test in the appropriate clinical situation is important, as not all pulp testing agents are suitable for all clinical situations. 

The aim of this paper was to review the literature regarding pulp testing of teeth. In order to identify relevant articIes, a PubMeD/Medline search was performed using the following keywords: dental pulp testing, sensibility testing, vitality test. Each keyword was subsequently searched using the “AND” function with each of the following other keywords: diagnosis, thermal, electric, laser Doppler flowmetry, and pulse oximetry. Further hand searching was then done using the reference lists in the articles identified.

## 2. Diagnostic Objectives of Pulp Testing

As an investigation, pulp testing can have several aims.

### 2.1. Assessment of Pulp Health Based on Its Qualitative Sensory Response

The assessment of pulp health based on its qualitative sensory response [1] is commonly done:

- prior to restorative, endodontic, and orthodontic procedures,
- as a follow-up and for monitoring the pulp after trauma to the teeth,
- in differential diagnoses, such as excluding periapical pathosis of pulp origin.

The most accurate way of evaluating the pulp status is by examination of histological sections of the tissue specimen involved to assess the extent of inflammation or the presence of necrosis as a means of gauging pulp health. Unfortunately in the clinical scenario, these are both impractical and not feasible; hence clinicians must use investigations such as pulp tests to provide additional diagnostic information.

#### 2.1.1. Pulp Vitality Testing, Pulp Sensibility Testing, and Pulp Sensitivity

**Pulp Vitality Testing: Assessment of the Pulp's Blood Supply**

Pulp tissue may have an adequate vascular supply, but is not necessarily innervated [4]. Hence, most of the current pulp testing modalities do not directly assess the pulp vascularity and this is exemplified by clinical observations [5] that traumatized teeth can have no response to a stimulus (such as cold) for a period of time following injury.

**Pulp Sensibility Testing: Assessment of the Pulp's Sensory Response**

Sensibility is defined as the ability to respond to a stimulus, [6] and hence this is an accurate and appropriate term for the typical and common clinical pulp tests such as thermal and electric tests given that they do not detect or measure blood supply to the dental pulp.

**Pulp Sensitivity: Condition of the Pulp Being Very Responsive to a Stimulus**

Thermal and electric pulp tests are not sensitivity tests although they can be used as sensitivity tests when attempting to diagnose a tooth with pulpitis since such teeth are more responsive than normal.

Clinicians performing pulp sensibility tests use the results, which are essentially qualitative sensory manifestations, to extrapolate and estimate the “vitality” and state of pulp health [1]. If the pulp responds to a stimulus (indicating that there is innervation), then clinicians generally assume that the pulp has a viable blood supply and it is either healthy or inflamed, depending on the nature of the response (with respect to pain, duration, and so forth), the history, and the other findings. 

The three types of responses can be summarised as follows.

- The pulp is deemed normal when there is a response to the stimulus provided by the sensibility test and this response is not pronounced or exaggerated, and it does not linger.
- Pulpitis is present when there is an exaggerated response that produces pain. Pulpitis can be considered as reversible or irreversible, depending on the severity of pain and whether the pain lingers or not. Typically mild pain of short duration is considered to indicate reversible pulpitis while severe pain that lingers indicates irreversible pulpitis [7, 8].
- The absence of responses to sensibility tests is usually associated with the likelihood of pulp necrosis, the tooth is pulpless, or has had previous root canal therapy.

It has been demonstrated that there is some association between the qualitative sensory manifestations and the histological appearance of the pulp [9]. However it is well demonstrated that this relationship is weak and inconsistent [10–12] at best, with false responses limiting direct extrapolation of pulp sensibility to pulp health and disease. 

### 2.2. Replication of Symptoms and Triggers for Pain Diagnostic Purposes

The replication of symptoms and triggers for pain diagnostic purposes [2, 13] is commonly done:

- to localise the source of pain,
- as an aid in excluding nonodontogenic orofacial pain.

In cases where an inflamed pulp is suspected to be the source of pain with the patient complaining of pain onset and aggravation by specific thermal triggers, pulp testing agents are useful in identifying the offending tooth [7, 14]. When the presentation of pain is inconsistent and atypical with the possibility of referred or nonodontogenic pain, pulp testing can assist in the correct diagnosis by a process of confirmation or elimination.

#### 2.2.1. Pulp Nociception Mechanism

Brännström's hydrodynamic theory [15] proposed that pulp pain is a result of nociceptors activated by fluid movement with possible other irritants through the patent dentine tubules [16]. The fast conducting myelinated A*δ*-fibres are known to be responsible for the acute “sharp shooting pain” whereas the slower conducting unmyelinated C-fibres are attributed to the “burning” pain with slower onset.

Often an anecdotal observation, using pulpitis resulting from rapidly progressing caries as an example, is that there seems to be more likelihood of having pain because there is less time for the dental pulp to react and protect itself by occluding the dentinal tubules [17]. This finding partly explains how the pain associated with pulpitis differs greatly in quality, severity, duration, onset, and trigger. It also explains how it can often be poorly localised [18]. 

With the progression of caries into dentine, the number of dentinal tubules being permeable is a major determinant to the degree of pain. The perceived pain also undergoes physiological modulation, with up or down regulation by inflammatory mediators which can be either endogenous or exogenous in origin. The changes in intrapulp pressure have a profound effect on sensory nerves of differing diameters, with the increase in pressure selectively blocking larger diameter A*δ*-fibres and activating the smaller C-fibres. As the pulp undergoes degeneration by the underlying disease, the C-fibres may still function since they are more resistant to hypoxia [1]. Once there is a total lack of response to a stimulus, it is likely that pulp necrosis will be well advanced [19]. 

It should also be noted that it is often difficult to clearly ascertain an accurate history of clinical symptoms due to the subjective nature of pain, the individual differences in pain threshold, and the mechanisms of pain modulation. Adding to the problem is the psychological difficulty for the patient in differentiating and communicating their pain to the clinician as well as the complex pathophysiology affecting nerve conductance not being fully understood by the profession [12]. 

## 3. Pulp Testing Techniques and Effectiveness

### 3.1. Pulp Sensibility Testing

#### 3.1.1. Thermal Tests

The application of agents to the teeth to increase or decrease temperature and to stimulate pulp sensory responses through thermal conduction has been the most commonly used modality for pulp testing. Whilst some of the techniques have been described as “crude” in many ways, their usefulness as diagnostic aids cannot be denied [2, 20].

**A. Cold Tests**

Ethyl chloride and ice have been popular in the past, [21, 22] but CO_2_ snow and other refrigerants such as dichlorodifluoromethane (DDM) have been shown to be effective [23] and superior [19] to ice and ethyl chloride.

**(i) Ice**

This is perhaps the simplest cold testing agent requiring practically zero cost to prepare and it can be made in a standard household freezer. A common way to make ice in useful sizes and dimensions involves freezing water in empty local anaesthetic cartridges. However the clinical handling, infection control issues, and the direct application of ice can be difficult and problematic [2]. Isolation with rubber dam may be of assistance to avoid thermal stimulation of multiple teeth.

**(ii) Refrigerant spray**

Due to its ease of storage, relatively cheap cost, and simple application technique, refrigerant spray is widely used in clinical settings. More effective agents such as DDM have superseded traditional refrigerants such as ethyl chloride. However, DDM, being a chlorofluorocarbon, has decreased in popularity and market availability due to environmental concerns of atmospheric ozone layer depletion [24]. Consequently, manufacturers have replaced DDM with other gases, including tetrafluoroethane (TFE) or a propane/butane/isobutane gas mixture stored in a pressurised can (Endo Frost, Roeko, Langenau, Germany). The application of the refrigerant spray requires a carrier such as a cotton pellet saturated with the substance prior to direct contact with the teeth as described by Jones [25]. Larger pellets, such as size 2, have larger surface areas than smaller cotton pellets, thus allowing better thermal conduction to occur between the carrier and the tooth being tested. Cotton buds with wooden handles and small cotton pellets have smaller surface areas available for contact and are therefore less efficacious in thermal conduction. Cotton rolls are generally not recommended as the portion with the dry fibre serves as a wick drawing all available refrigerants away from the tooth structure.

**(iii) Carbon dioxide snow (CO_2_)**

CO_2_ snow, or dry ice, is prepared from a pressurized liquid CO_2_ cylinder [26] using a commercially available apparatus known as the Odontotest (Fricar A.G. Zurich, Switzerland). This involves the liquid CO_2_ being forced through a small orifice such that when it comes under atmospheric pressure most of the liquid will be converted into dry ice. The CO_2_ is collected in a hollow removable carrier, encased in a thin plexiglass tube. The dry ice is collected in a “pencil stick” form that can then be applied to one tooth at a time with the aid of the supplied plunger.

**Rate of Temperature Decrease and Speed of Pulp Response**

Recorded in vitro temperatures for DDM (−50°C), Endo Frost (−50°C), and TFE (−26°C) are all higher than CO_2_ (−78°C) [24]. However, CO_2_ is not as cold when used clinically, [27] where it has been reported to be −56°C, similar to DDM which is known to have a temperature as low as −50°C [19, 28]. The in vivo temperature for Endo FrostTM is approximately −28°C whilst TFE is −18.5°C [28]. Some in vitro studies [19, 29] have shown that CO_2_ produces a slightly larger decrease in temperature in a short period of time, especially with metallic restorations (such as amalgam and gold restorations) which allow better thermal conduction. Another in vitro study [24] reported that refrigerants such as TFE (or similar) decrease temperature by 1-2°C more than CO_2_ within a 10-second timeframe. In the same study [24], it was found that teeth restored with metal crowns (such as full gold or porcelain fused to metal crowns) had faster rates of temperature decrease when tested with either refrigerant sprays or CO_2_ compared to teeth restored with full ceramic crowns. Refrigerant sprays have also been shown to evoke faster pulp responses by one to three seconds in vivo [19, 23] without any differences in accuracy. This could be attributed to the larger surface areas of the refrigerant being in contact with the tooth because of the size of the cotton pellet carrier [25] used. Whether the higher rate of change in temperature of 1-2°C in 1–3 seconds is clinically relevant is doubtful [23, 24]. When testing multiple teeth, such as the whole dental arch, CO_2_ is more convenient as the rate of dissipation is much lower than that of any of the other refrigerants [24].

**Safety Concerns of Cold Tests**

Concerns have been raised in the past about the possible damaging effects of cold testing agents with particular reference to CO_2_ given its measured laboratory temperature of −78°C. Ehrmann [2] described the “phenomenon of Leidenfrost” that occurs when a small amount of CO_2_ snow enters the oral cavity but causes no harm in spite of its physical contact with the oral mucosa. This is due to an insulating layer of gaseous CO_2_ surrounding the melting mass as the dry ice “film boils” during sublimation so there is insufficient time for soft tissue burns to occur.

Lutz et al. [30] found that cracks may be formed on enamel surfaces from direct CO_2_ snow contact. Subsequently, to address this issue, a three part series of studies was reported by Peters et al. [27, 31, 32]. In addition, both the in vitro and in vivo effects of exposure of low temperatures to the pulp and to the tooth structure were evaluated. The first part of the study [31] was an in vitro experiment where CO_2_ snow was applied for two minutes to 15 human teeth. Nine of the fifteen teeth were noted to have pre-existing fissure and/or craze lines. Only one of the nine teeth had increased fissure size after application of the CO_2_ snow.

The second part of the study [32] was an in vivo study undertaken on dog teeth with CO_2_ snow being applied to each tooth for up to two minutes. The animals were subsequently sacrificed. Half of the teeth were tested with CO_2_ snow two days prior to sacrifice of the animals, and the other half were tested 51 days prior to sacrifice. Histological sections of the pulp and scanning electron microscopic (SEM) evaluation of the dental hard tissue specimens postmortem showed no signs of damage in all samples.

The third part of the study was similar [27] but it was performed on humans. A two-minute application of CO_2_ snow was carried out on ten human teeth scheduled for extraction. Results were similar to the animal study and the SEM evaluation of the replicas/impressions of the specimens showed no physical damage.

Fuss et al. [33] also conducted combined in vivo and in vitro studies placing CO_2_ snow on teeth scheduled for extraction. The findings were similar to the Peters et al. studies [27, 31, 32]. The in vitro portion of the study also showed that CO_2_ snow only decreased the pulp temperature by less than 2°C after five seconds of application—a change that is not sufficient to damage the pulp, as Frank et al. [34] estimated that pulp tissue is only irreversibly damaged after being frozen at approximately −9°C. In vitro measurement of interproximal thermal conduction between natural teeth has shown that less than 0.25°C is transferred in this manner [29]. This is likewise insignificant and not a safety concern.

**B. Heat Test**

Typical methods used include gutta percha [1] or compound material heated to melting temperature and directly applied to the tooth being tested with lubricant in order to facilitate removal of the material. Heated ball-ended metallic instruments [35] placed near the tooth (but without touching the tooth surface), battery-powered controlled heating instruments such as Touch n' Heat and hot water bathing [7] with the tooth isolated by rubber dam are other alternative methods.

**Safety Concerns of Heat Tests**

The temperature of melting gutta percha used in pulp testing is approximately 78°C [33] but it has been reported to be up to 150°C [1]. Zach et al. [36] noted that an increase of 11°C that occurs during restorative procedures without adequate cooling can harm the pulp. Therefore, prolonged contact with heat is a safety concern. In the in vitro portion of the Fuss et al. [33] study, it was shown that heat testing using gutta percha in the manner described above increased pulp temperature by less than 2°C with less than five seconds of application—a temperature change that is unlikely to have caused pulp damage.

**Accuracy of Thermal Test**

Mumford [37] compared heat and cold pulp sensibility tests using ethyl chloride and heated gutta percha on anterior teeth and premolars that had clinically healthy pulps in both adults and children. It was found with both ethyl chloride and heated gutta percha that most of the premolar teeth failed to respond, especially in adults. Furthermore, cold testing was found to be more accurate than heat in the same experiment. Dummer et al. [12] had comparable results with thermal testing of clinically healthy pulps, but found that in teeth diagnosed with irreversible pulpitis, heated gutta percha was no more effective than ethyl chloride.

Even with the proven effectiveness of CO_2_ and refrigerant sprays, false responses are nonetheless possible. False-positive responses have been reported with cold tests applied on endodontically treated teeth, [23] and this has been attributed to these particular teeth having been restored with cast metal crowns so the transference of the intense cold could stimulate either the gingivae or the adjacent teeth, or both of these. Instances have been reported where no response has been noted in teeth with a healthy pulp in elderly patients, [38] in heavily restored teeth, [1] particularly in the older population, and in teeth with a history of trauma [5]. Teeth in all of these situations would be expected to have a reduced size of their pulp chambers due to the extra deposition of dentine that occurs, whether this is physiological, reactionary, or reparative in nature. This results in the sensory elements of the pulp being well insulated against changes in temperature and hence there is a higher chance of false test results.

#### 3.1.2. Electric Pulp Test

Electric pulp testing (EPT) works on the premise that electrical stimuli cause an ionic change across the neural membrane, thereby inducing an action potential with a rapid hopping action at the nodes of Ranvier in myelinated nerves [7]. The pathway for the electric current is thought to be from the probe tip of the test device to the tooth, along the lines of the enamel prisms and dentine tubules, and then through the pulp tissue [20]. The “circuit” is completed via the patient wearing a lip clip or by touching the probe handle with his/her hand; alternatively, the operator can have one “gloveless” hand that touches the patient's skin [39, 40]. A “tingling” sensation [41] will be felt by the patient once the increasing voltage reaches the pain threshold, but this threshold level varies between patients and teeth, and is affected by factors such as individual age, pain perception, tooth surface conduction, and resistance [20].

The correct technique for using the electric pulp tester is also important for accurate responses. In order to ensure that the appropriate current pathway is followed, correct placement of the EPT probe tip flat against the contact area, and having a conducting medium such as toothpaste between the probe tip and the tooth surface is essential [42]. Jacobson found in an in vitro experiment involving incisors and premolars, that placing the probe tip labially within the incisal or occlusal two-thirds of the crown gave more consistent results [43].

**Safety Concerns of EPT**

In EPT operation manuals, warnings have been made that the current produced by the testing device may cause danger to patients who have cardiac pacemakers, with the risk of precipitating cardiac arrhythmia via pacemaker interference. This concern is based on a sole animal study [44] where EPT interfered with a pacemaker fitted in a dog. At the time of that study (the early 1970s), cardiac pacemakers were primitive but as pacemakers have become equipped with better shielding, more recent studies have shown no interference from EPT or similar electrical dental devices [45–47].

**Accuracy of EPT**

Electric pulp tests are known to be unreliable in many instances, producing false results in healthy immature teeth [7, 48] with incompletely formed roots which may be erupting since these teeth may take up to five years before the maximum number of myelinated fibres reaches the pulp-dentine border at the plexus of Rashkow. This is also when apical root maturation occurs. Teeth with pulp canal calcification (PCC) and patients suffering from primary hyperthyroidism frequently have an increased sensory response threshold to EPT. In the case of PCC, the sensory response may be completely blocked, whereas hypercalcaemia from hyperthyroidism may require twice as much current as that which is normally needed to elicit a response from a clinically normal pulp [49].

False results are also possible in teeth with healthy pulps undergoing orthodontic treatment [50] because the pulp's sensory elements may be disturbed for up to nine months. Similarly, recently traumatised teeth undergoing pulp repair may also have false results and thus may not respond to EPT [7].

In humans, many clinical observations from dental trauma studies [51, 52] have indicated that it can take pulps a minimum of 4–6 weeks following trauma for sufficient recovery of sensation to obtain valid pulp testing results. Theories proposed by Öhman [51] for this loss of pulp sensibility include pressure or tension on the nerve fibres, blood vessel rupture, and ischaemic injury. It is then assumed that these effects were reversible in the cases where the pulp sensation recovered.

Pileggi et al. [53] have shown in ferrets that 10–12 days is required for the sensory component of the pulp to start to respond EPT again as damage from trauma heals. It has also been observed that hyperaemia and/or transient nerve decompression may be responsible for the temporary sensory loss.

Inaccurate responses are known to occur with EPT when the current is conducted to adjacent teeth [54], for example, when two adjacent teeth have contacting proximal metallic restorations. Periodontal tissues, breakdown products from pulps undergoing necrosis, and remnants of inflamed pulp tissues may also cause sensory stimulation leading to false responses [38, 55, 56].

#### 3.1.3. Test Cavity

The preparation of a test cavity has been suggested as a last resort in a tooth where no other means can ascertain the pulp status [1, 2]. Cutting into dentine using a high or low speed bur without local anaesthetic may give some indication of whether the sensory element of the pulp is still functioning although it is unlikely that this procedure would provide any more information than thermal and electric pulp sensibility tests. Whilst the defect made in the tooth can be repaired with restorative dental materials, this method is nonetheless considered invasive and irreversible. A consideration must be made for the apprehensive patient, as it is likely that he or she may react nervously and confound the response. Hence, test cavities are not generally recommended as a means of testing pulp sensibility.

## 4. Pulp Vitality Testing

### 4.1. Laser Doppler Flowmetry (LDF)

Research into the application of laser Doppler flowmetry to traumatized teeth has been extensive [57–64]. Other applications have been reported in paediatric dentistry, [65] as an aid in the differential diagnosis of nonodontogenic periapical pathosis [66] and to assess pulp blood flow [67]. The aim of this technique is to objectively measure the “true” vitality of the pulp (i.e., the pulp blood flow rather than its sensory function) without invasive procedures [68] such as intravital microscopy and gas desaturation. 

The laser Doppler flowmetry technique was first described in dental literature in 1986 by Gazelius et al. [69] This electro-optical technique uses a laser source that is aimed at the pulp, and the laser light travels to the pulp using the dentinal tubules as guides [70]. The backscattered reflected light from circulating blood cells is Doppler-shifted and has a different frequency to the static surrounding tissues. The total backscattered light is processed to produce an output signal [55]. The signal is commonly recorded as the concentration and velocity (flux) of cells using an arbitrary term “perfusion units” (PU) [70, 71], where 2.5 volts of blood flow is equivalent to 250 PU [72]. In order to record the Doppler shift of the blood cells, both the probe and tooth need to be completely still. Hence, a stabilising splint made of polyvinyl siloxane (PVS) or acrylic is usually used [70].

**Technical Considerations of LDF**

Various studies have been performed to investigate the preferred testing parameters for laser Doppler flowmetry. The laser beam produced is a low power beam ranging from 1-2 mW. Different wavelength lasers can be produced by different sources: 633 nm through Helium-neon laser, or 780 and 810 nm by semiconductor diode lasers [73]. The initial study by Gazelius et al. [69] found that a 750 nm laser penetrated deeper but was associated with signal contamination of non-pulp origin from surrounding tissues. Odor et al. [74] found that laser light sources with longer wavelengths had better sensitivity to moving red blood cells due to deeper penetration into the pulp vasculature. However, later studies [75–77] noted the extensive scattering of light with longer wavelength lasers contributing to the problem of signal contamination.

Different ranges of bandwidth can be set to filter the reflected signal, with a wider frequency being more sensitive to moving red blood cells with a wider range of velocity [78]. Theoretically, a wider bandwidth, such as 15 kHz, is preferred but there is some evidence that the narrower 3 kHz bandwidth may be a more ideal filter bandwidth for pulp testing [74, 75].

The end of the LDF probe which contacts the tooth contains both sending and receiving optic fibres, with one of the configurations being one source and two detectors in a triangular arrangement at the probe end [55]. Calibration of the probes is important to ensure accurate readings [79]. The larger the optical fibre separation distance on the probes, the higher the signal output as a larger surface area is covered, and also potentially a higher chance of blood flow signal contamination from non-pulp sources [80]. To date, 0.5 mm or 0.25 mm separation distances seem to be preferred in experiments [55, 75].

Due to the pulsatile nature of blood flow, many studies [55, 65, 67, 81] have observed that LDF recordings in teeth with intact pulp blood flow have rhythmic fluctuations or oscillations. Synchronisation was found with both heartbeat and electrocardiogram (ECG) readings when taken simultaneously. In teeth without pulp blood flow however, usually only irregular fluctuations can be observed in contrast to the concurrent ECG readings.

**Limitations of LDF**

Unfortunately, contamination “noise” due to backscattered light from the periodontal tissues [82] is at present impossible to completely eliminate in LDF, [69, 76] even if a covering such as a PVS splint is applied [75]. It has been observed that the closer the probe is positioned to the gingival margin, the higher the signal output will be (due to greater pulp tissue volume) [83] but the potential gingival contamination is also higher [70]. Studies suggest that 2-3 mm from the gingival margin is the ideal position for the probe tip as this creates a balance between minimizing the noise and having a recognizable signal volume [71, 75].

Notwithstanding the above, Soo-Ampon et al. [84] found that up to 80% of the LDF output signal in human incisors may be non-pulp in origin if attempts at tooth isolation are not made. These authors observed that humans have a smaller pulp-chamber-to-crown ratio, and hence there is a relatively high proportion of non-pulp signal in comparison to LDF readings taken from animals such as pigs and cats. Polat et al. [85] compared teeth that had undergone a pulpectomy with contralateral healthy pulps as controls. They also found that approximately 70% of the LDF readings from teeth with the pulps removed were non-pulp in origin.

In another study, Polat et al. [77] examined the scattering and penetration properties of the laser used in LDF by using a camera with slow speed shutters. They demonstrated that the laser can densely penetrate up to 4 mm in depth and less densely for up to 13 mm. This also suggests that even with proper isolation of the tooth, some signal contamination from the periodontium is inevitable. They also demonstrated that without isolation, the laser light can scatter from the source tooth to the whole oral cavity which also can potentially contribute to signal contamination.

It is impossible to calibrate the blood flow in absolute units with LDF techniques since the output is not linearly related to blood flow. That is, when the output signal increases 100 percent, the blood flow may not increase by 100 percent [70]. Thereby the LDF values recorded must be interpreted with care.

Any obstruction and/or interference of the light pathway can render LDF useless—examples being restorations, especially if they are full coverage crowns. Teeth in some ethnic groups may have different pigments in the tooth structure that may also affect the scattering and filtering of light [84]. Optical properties of root-treated teeth may differ from teeth with intact pulps [75, 84]. Heithersay and Hirsch [86] reported a case where a traumatised upper central incisor had staining following trauma and subsequently returned to normal colour. It was noticed that the LDF readings were zero while the tooth was discoloured by blood products but the LDF readings later returned when the tooth stain disappeared. A similar observation was made in the same study with a different patient who had a bruise under a fingernail

**Accuracy of LDF as a Pulp Test**

There have been differing views with regards to the accuracy of pulp testing using LDF, given that false results suggesting no blood flow are possible when the laser pathway is interfered with or obstructed [84, 86]. Likewise, the amount of signal contamination from non-pulp sources, [84, 85] primarily the periodontium, can lead to false readings suggesting the presence of pulp blood flow.

At times, the interaction between these factors can lead to interesting results. Ingólfsson et al. [55] observed that two teeth with clinically diagnosed pulp necrosis gave higher LDF readings than normal pulps from control teeth. The clinical diagnoses were confirmed during subsequent endodontic treatment. Both of these teeth had increased amounts of dentine in the crown, with one having “complete radiographic calcification” of the pulp space, whilst the other had a significantly smaller pulp chamber space when compared to the patient's other teeth. It was assumed by the authors that the higher output signals were a result of the teeth having altered optical properties and conducting light to the blood vessels in the periodontal ligaments.

In order to examine intraindividual differences, Ramsay et al. [83] took repeated pulp blood flow readings of thirteen upper central incisors at the same site over four different test sessions, at approximately two-week intervals and with two sets of readings per tooth per session. It was found that the intrasession variations of the recordings were minor but the intersessional differences were statistically significant. This was similar to the Gazelius et al. study [87] which found 2%–14% intraindividual variation when testing sessions were two months apart. Given the experimental conditions, it was postulated that the inconsistency was due to intrinsic variations of pulp blood flow and the inability to place the probe in exactly the same position at each of the test sessions despite using customized acrylic splints for all subjects.

Norer et al. [88] investigated both interindividual and intraindividual pulp blood flow characteristics for different types of maxillary teeth over three sessions at seven day intervals but only one reading per tooth was taken per session. The intraindividual difference between sessions was found to be small, in contrast to the findings of Ramsay et al. [83] and Gazelius et al. [87]. There was an exception with first molars where intraindividual differences were found, and this was attributed to technique-related difficulties with probe alignment with respect to both the tooth location and the pulp chamber anatomy.

**Interpretation of Pulp Status Using LDF**

It is common for the pulp status of a tooth in question to be determined by measuring flux values [57, 58, 89] and comparing them to a control tooth of some description. However, in the light of problems with signal contamination, differentiating intact pulp blood flow or the lack thereof becomes a challenge. A contralateral tooth from separate subjects or from an area not affected by intervention/testing has been recommended as an appropriate control for LDF [83]. With the exception of maxillary central incisors, interindividual differences of LDF were significant in Norer et al. study [88]. An average of the pulp blood flow readings for identification of a healthy pulp is therefore impractical, if not impossible, to achieve. Intraindividual differences are more likely to be less, such that simultaneous LDF readings can be taken in the same patient from both a suspect tooth and a tooth with a known healthy pulp, preferably using a tooth of the same morphological type such as the contralateral tooth [75, 89].

Roebuck et al. [75] investigated 11 sound teeth (contralateral controls) and 11 teeth with pulpless and infected root canal systems using a laser Doppler flowmeter. All 22 teeth were maxillary anterior teeth from 11 subjects whose ages ranged from 9 to 16 years. Both 633 nm and 780 nm laser sources were used with different filter frequencies and varied probe-end optic fibre separation distances. PVS splints were used and the probes were placed 2-3 mm from the gingival margin. Overall, the pulpless teeth were found to have, on average, 37.3% less output signals than the sound teeth.

Ingólfsson et al. [55] investigated 22 teeth in a manner similar to Roebuck et al. [75]. Nine patients with ages ranging from 11–37 years were tested, and only a 633 nm laser source was used. The overall signal output for pulpless teeth was found to be, on average, 42.7% lower than the sound teeth, which was not dissimilar to the findings of Roebuck et al. [75].

Therefore, whilst the pool of data from the two studies is quite small and the testing parameters differ slightly, it appears that a tooth with at least 40% less LDF output signal ((diseased pulp flux/healthy pulp flux) < or = 0.6) may warrant further clinical and radiographic investigation with regards to the status of the pulp. Software assisted fast Fourier transforms have also been advocated as an adjunct to identify pulp blood flow, and this is done mathematically by matching LDF output signal frequency to references such as heart beat and/or slow wave vasomotion [74].

### 4.2. Pulse Oximetry

Compared to laser Doppler flowmeters, pulse oximeters are relatively inexpensive [90, 91] and commonly used in general anaesthetic procedures. The term oximetry is defined as the determination of the percentage of oxygen saturation of the circulating arterial blood [92]. Oxygenated haemoglobin and deoxygenated haemoglobin are different in colour and therefore absorb different amounts of red and infrared light. The pulse oximeter therefore utilises probes emitting a red and an infrared light to transilluminate the target vascular area, which allows the photodetector to identify absorbance peaks due to pulsatile blood circulation, and thereby calculate the pulse rate and oxygen saturation level (SaO_2_) [91, 92]. 

An in vitro study by Noblett et al. [91] compared pulse oximetry with blood gas saturation in a simulated pulp blood flow model and showed promising results. Initial in vivo trials of pulse oximetry on ten adults by Kahan et al. [90] found poor results with the prototype oximeter unable to obtain correct readings for clinically healthy pulps. However, a pilot study by Goho [93] found that 48 permanent and deciduous teeth had SaO_2_ on average in the range of 93–94% in comparison to the SaO_2_ taken from index fingers, which is approximately 97%. Radhakrishnan et al. [92] reported registering SaO_2_ of 100 permanent teeth of children in the region of 80%. It is interesting to note that both studies had ten root-filled teeth as controls, all of which recorded 0% SaO_2_. The lower SaO_2_ and discrepancies in the values obtained in the two studies were attributed to differing optical properties of the teeth because infrared light undergoes diffraction when passing through teeth [94] and scattering of the light rays as they pass through the gingivae [95].

### 4.3. Other Noninvasive Experimental Tests

- Photoplethysmography [94, 96]is an analysis of the optical property of a selected tissue. It was developed for pulp testing in an attempt to improve pulse oximetry, by adding a light with a shorter wavelength. The results, whilst promising, were nonetheless equivocal.
- Spectrophotometry, using dual wavelength lights in an effort to ascertain the contents of enclosed spaces such as the pulp chamber, has been tested with optimistic, but only initial, experimental results [97].
- Transmitted Laser Light (TLL) [98, 99] is an experimental variation to LDF, aimed at eliminating the non-pulp signals. TLL uses similar sending/receiving probes as conventional LDF, but the probes are separate. Thus the laser beam is passed through from the labial or buccal side of the tooth to the receiver probe which is situated on the palatal or lingual side of the tooth. The limitations with TLL are the same as with any laser technology where obstruction and/or interference from within the tooth structure will affect the results.
- Transillumination [100] utilises a strong light source which identifies colour changes that may indicate pulp pathosis. This technique may not be useful in large posterior teeth and especially in teeth with large restorations. However, it is a helpful adjunct to conventional pulp tests and it can help to identify cracks in teeth.
- Ultraviolet light photography [101] examines different fluorescence patterns that may allow additional contrast of otherwise more difficult to observe visible changes. It has similar limitations as transillumination, and it is only an adjunct to conventional pulp tests, at best.
- Surface temperature measurement [102–104] has not found practical clinical use in pulp testing, even though there have been reports that a measurable temperature difference can be found over time in teeth with healthy pulps in contrast to teeth with diseased pulps. Potential interfering factors such as breathing by the patient and the lengthy time required for this technique are the major drawbacks.

## 5. Comparative Studies of Pulp Testing

### 5.1. Comparisons between EPT and CO_2_

Fuss et al. [19] compared the accuracy of EPT with cold testing agents including DDM, CO_2_, ethyl chloride and ice. Ninety-six maxillary central incisors from 24 patients were all diagnosed with clinically healthy pulps. Half of the patients were adults and the others were children less than 13 years of age. Overall DDM and CO_2_ had accuracies approaching 100 percent, and EPT approaching 90% with no statistical difference between these three testing modalities. These three tests all performed better (*P* < .01) than ice and ethyl chloride both of which had less than 50% accuracies. The results for the 12 adults on the other hand showed EPT accuracy approached 100% and both DDM and CO_2_ approached 95%, but there were no statistically significant differences between the three testing modalities. It was noted that in the child participants, CO_2_ and DDM accuracy approached 100%, which was similar to the overall results. However, the accuracy of CO_2_ and DDM in children was significantly higher (*P* < .05) than EPT, which only scored approximately 75%. 

These findings were consistent with the observations made by Fulling and Andreasen [48] who found that there are more false negative results with EPT in erupting teeth/incompletely formed roots until the closing of the apical foramen. This is in contrast to the more consistent results given by CO_2_ in the Fulling and Andreasen study [48]. They also noted that CO_2_ was more reliable in traumatised teeth.

#### 5.1.1. Comparison between False Responses of EPT and CO_2_

Peters et al. [38] investigated 1488 anterior and posterior teeth in 60 adult patients with either healthy or diseased pulps and periapical tissues. Positive responses and lack of responses to sensibility tests were analysed using CO_2_ and EPT. Particular emphasis was placed on having the gingival/mucosal tissue response as a control when using EPT, since false positives were likely when the EPT voltage level that was needed to evoke that a response from a suspect tooth was lower than that needed to stimulate the gingival tissue. However, this was not always the case as it was observed that teeth without a healthy pulp could respond at levels lower than the mucosal tissue.

Peters et al. [38] also reported that if a tooth did not respond to both CO_2_ and EPT, plus it had no history of trauma, then the pulp status was almost certain to be diseased. If the tooth did not respond to CO_2_ but did respond to EPT at a value higher than the mucosal tissue response, then a diseased pulp was possible, and further clinical and radiographic investigation was recommended. If the tooth responded to CO_2_ but did not respond to EPT, then the EPT result was more likely to be faulty.

### 5.2. Comparison of Heated Gutta Percha, Ethyl Chloride, and EPT

Petersson et al. [105] evaluated cold and heat tests as well as EPT by calculating the relevant sensitivity, specificity, positive predictive value, negative predictive value, and accuracy for each test with a given disease prevalence. Responses were measured from 75 anterior and posterior teeth with healthy and diseased pulps from 65 patients whose ages ranged from 21 to 79 years. These teeth were investigated according to whether they were true/false negatives (TN, FN where “negative” was defined as the absence of disease) or true/false positives (TP, FP where “positive” was defined as the presence of disease).

When discussing studies about pulp testing, the sensitivity of a pulp test can be defined as its ability to identify teeth with no pulp or with a diseased pulp (Sensitivity = TP/(TP + FN), and this should not be confused with *pulp sensitivity,* as described earlier. The specificity of a pulp test is its ability to identify pulps without disease (Specificity = TN/(TN + FP)). The positive predictive value is the probability that a “positive” result truly represents a tooth with a diseased pulp or with no pulp (Positive predictive value = TP/(TP + FP)) and the negative predictive value is the probability that a “negative” result truly represents a disease free pulp (Negative predictive value = TN/(TN + FN)). Under the same definition, the overall rate of agreement of the test results to the actual pulp health can be expressed as accuracy (Accuracy = (TP + TN)/(TP + TN + FP + FN)). The results of the tests from Petersson et al. [105] are summarised in Table 1.

### 5.3. Comparison of LDF, Ethyl Chloride, and EPT

Evans et al. [89] examined 67 traumatised anterior teeth that had either necrotic pulps or pulpless and infected root canal systems in 55 patients whose ages ranged from 8–34 years, and 84 teeth with healthy pulps in 84 patients whose ages ranged from 7–34 years using LDF, EPT, and ethyl chloride. The LDF test used a 633 nm laser source, 4 kHz filter bandwidth, and a PVS splint to stabilise the probe which had 0.5 mm optic fibre separation. The probe was placed 2-3 mm from the gingival margin. Their results are summarised in Table 2.

LDF had the highest sensitivity and specificity in this study compared with the other pulp tests. The interpretation of the LDF readings was done by comparing the mean and range of flux as well as the relative percentage differences expressed as ratios. However, the authors commented that the LDF test was very technique sensitive and time consuming.

#### 5.3.1. Validity of Cross-Study Comparisons

Cross interpretation between studies comparing the performance of pulp tests needs to be approached with caution. It is important to note that the results can only be directly compared with another data set where the disease prevalence is the same [89]. Petersson et al. [105] also noted that study designs vary: Fuss et al. [19] examined both sensitivity and specificity, whilst Peters et al. [38] examined sensitivity only, and neither study analysed the positive and negative predictive values. All of these studies had variations in participants' age and teeth, with Evans et al. [89] and Fuss et al. [19] examining anterior teeth in children and adults whilst Petersson et al. [105] and Peters et al. [38] examined anterior and posterior teeth in adults only. These factors may partly explain the variations observed in their results.

## 6. Summary

Pulp sensibility testing, even with its limitations, has been and still remains a very helpful aid in endodontic diagnosis. Attempts at measuring the true pulp blood flow clinically have had mixed success, with laser Doppler flowmetry being one of the popular techniques applied in dental traumatology. Currently, no vitality tests have been proven to be superior in all aspects compared to pulp sensibility tests. Further research is needed to improve the reliability and accuracy of diagnostic dental pulp tests.

## Figures and Tables

**Table 1:** Pulp test results from the Petersson et al. [105] study. The disease prevalence in this study was 39%. Note: the heat test with gutta percha recorded a higher number of false positives compared to ethyl chloride and EPT.

	Ethyl Chloride	Heated Gutta Percha	EPT
Sensitivity	0.83	0.86	0.72
Specificity	0.93	0.41	0.93
Positive predictive value	0.89	0.48	0.88
Negative predictive value	0.90	0.83	0.84
Accuracy	0.86	0.71	0.81

**Table 2:** Pulp test results from the Evans et al. [89] study. The disease prevalence in this study was 44%.

	Ethyl Chloride	LDF	EPT
Sensitivity (approximately)	0.90	1.00	0.85
Specificity (approximately)	0.88	1.00	0.95
